# Performance of a Diaphragmed Microlens for a Packaged Microspectrometer

**DOI:** 10.3390/s90200859

**Published:** 2009-02-06

**Authors:** Joe Lo, Shih-Jui Chen, Qiyin Fang, Thanassis Papaioannou, Eun-Sok Kim, Martin Gundersen, Laura Marcu

**Affiliations:** 1 Biomedical Engineering, University of Southern California, Los Angeles, CA, USA; E-Mail: jlo@nano.com; 2 Electrical Engineering and Electrophysics, University of Southern California, Los Angeles, CA USA; E-Mails: shihjuic@usc.edu; eskim@usc.edu; mag@usc.edu; 3 Cedars Sinai Medical Center, Los Angeles, CA, USA; E-Mail: p_thanassis@yahoo.com; 4 Engineering Physics, McMaster University, Hamilton, Ontario, Canada; E-Mail: qiyin.fang@mcmaster.ca; 5 Biomedical Engineering, University of California at Davis, USA

**Keywords:** Microoptics, microlens, MEMS, spectroscopy, Gaussian propagation

## Abstract

This paper describes the design, fabrication, packaging and testing of a microlens integrated in a multi-layered MEMS microspectrometer. The microlens was fabricated using modified PDMS molding to form a suspended lens diaphragm. Gaussian beam propagation model was used to measure the focal length and quantify M^2^ value of the microlens. A tunable calibration source was set up to measure the response of the packaged device. Dual wavelength separation by the packaged device was demonstrated by CCD imaging and beam profiling of the spectroscopic output. We demonstrated specific techniques to measure critical parameters of microoptics systems for future optimization of spectroscopic devices.

## 1. Introduction

Miniaturized and microscaled spectrometers can enable and improve many applications of fluorescence based detection, including minimally invasive diagnostic and surgical techniques [1,2]. The majority of current miniaturized spectrometers are simple spectrograph designs, requiring only a fixed reflective grating and a linear detector array coupled with fixed optics. These grating-based systems have been further applied to micro-(opto)-electro-mechanical system, M(O)EMS-based tunable laser devices [3]. Furthermore, Bragg reflector or Fabry-Perot based filters have been applied to fiber based wavelength selectable devices [4]. However, spectrographs with photodetector arrays, though easier to miniaturize mechanically, cannot utilize high-speed/sensitivity photomultiplier (PMT) detectors that are more straightforwardly implemented in a monochromator [5-7]. Moreover, filter based systems require large arrays and are sensitive to surface quality and reflectivity, making tunable MEMS integration a challenge. Thus, for high speed/sensitivity application such as time-resolved fluorescence, a tunable grating monochromator is the best spectrometer design for miniaturization.

We have developed a microspectrometer based on a vibrating grating and microlenses in order to utilize high speed PMT in a microscale probe. In this microspectrometer, the microlenses greatly influence the dispersion performance, thus its fabrication and characterization is the main theme of this paper. Typically, miniaturized systems such as our MOEMS microspectrometer will be limited by spectral resolution due to extremely small distances available to disperse light. However, as time-resolved fluorescence occurs over a broader spectral window while utilizing the additional dimension of time information for detection, the microlens-enabled MOEMS microspectrometer is the most sensible solution that uniquely addresses the needs of a miniaturized time-resolved detection platform.

**Figure 1.**
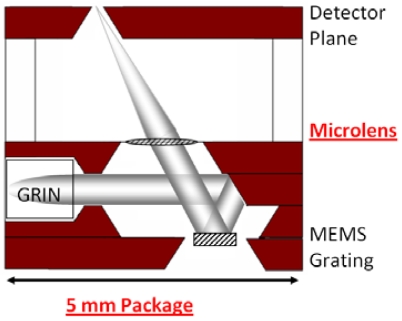
Cross-sectional view of the microspectrometer, showing the 5 mm silicon packaging with microlens, the MEMS grating, and other optical components.

We use microfabrication in order to realize the microspectrometer, based on monochromator design. It is a multi-layered system comprised of 1) 250 l/mm MEMS grating for wavelength tuning, 2) diaphragmed microlens, and 3) a wafer-level encapsulation process for packaging (Figure 1). Based on a Czerny-Turner monochromator modified with a Gradient Index (GRIN) collimator input and microlens focused output, the dispersion related performance is heavily dependent on the microlens capabilities. The GRIN coupled fiber is fabricated in-house with a 50 μM multimode fiber butt coupled with a 1.8 mm diameter 0.25 GRIN lens (Thorlabs and Newport), epoxy bonded to a glass pipette tube. Its divergence is around 1.8 degrees at 2 cm. As the diffracted rays from the actuated grating traverses the multi-layered packaging, the microlens translates the angularly dispersed wavelengths into focal positions at the exit plane, where a physical slit sits before the detector. Because of this crucial role in angle-to-position translation, the diaphragmed microlens has significant performance impact on the microspectrometer.

A number of fabrication techniques have been developed for microlens in imager arrays, waveguide coupling, endoscope microoptics, and biologically inspired structures [8-11]. General classes of passive microlens fabrication include 1) lithograph patterned material reflow, 2) diffractive microlens, and 3) mold formation. The reflow of thick AZ photoresist is a well-explored method for producing spherical profiles of patterned structures [12]. However, reflown resist microlenses are supported on opaque bulk substrates and cannot perform as a suspended lens. Diffractive microlenses produce artifacts and unnecessarily decrease transmittance [13]. Material molding presents the best method to form a suspended microlens for our microspectrometer. We use a modified molding method similar to other recent studies [14] to realize a unique suspended microlens for our system.

## 2. Diaphragmed Microlens Fabrication and Packaging

### Microlens

**Figure 2.**
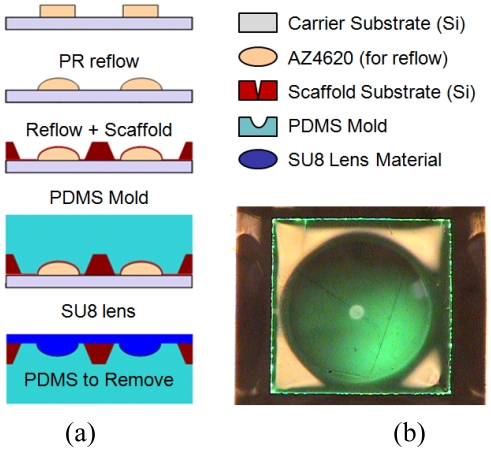
(a) Process flow of the microlens starts from photoresist reflow and is realized through soft-lithography molding. (b) Photomicrograph of a finished microlens, top down.

The microlens was fabricated of polydimethylsiloxane, PDMS via soft-lithography using a molding technique (Figure 2a). First, a pattern of circular disks (1.2 mm diameter) was transferred to a double-layer AZ4620 photoresist on silicon substrate in preparation for the designed lens reflow.

Second, the double-layer resist was reflown into a semi-spherical profile via contact hotplate heating at 145°C. Third, the lens mount wafer, fabricated earlier, was bonded to the lens substrate via thin coat of AZ5214-E. Next, premixed and degassed (via centrifugation at 3.5 kRPM) PDMS was cured over the wafer complex through gradual curing from 60-90°C for 30 minutes. The thick, AZ4620 substrate was separated from the rest of the device via ultrasonication in acetone. Finally, 50 μm thick SU8 was spun onto the mold, exposed, and cross-linked slowly from 60-90°C for 1.5 hrs. The resultant SU8 lens had a refractive index of 1.6 with curvature calculated by volume conservation before and after the reflow. The PDMS layer was then subsequently removed. The final structure was a robust, cured-epoxy diaphragm-suspended microlens with controlled curvature (Figure 2b).

### Microspectrometer Assembly

**Figure 3.**
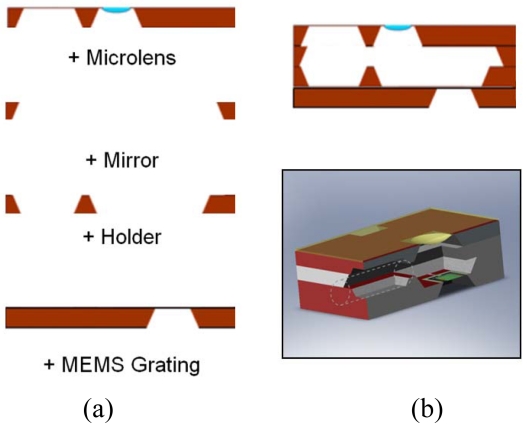
(a) Multi-layer silicon cavity packaging of microlens and other MEMS components of the micro-spectrometer. (b) The 5 mm by 5 mm package with cross-section (top) and perspective (bottom) view.

The MEMS grating and fiber input components were fabricated separately. To package the lens with wafer-level encapsulation, multilayers of silicon substrates with cavity geometries were fabricated. The individual silicon substrate layers were fabricated by KOH bulk microfabrication. First, 7,000 Å thick layer Si_x_N_y_ thin film was deposited by low pressure chemical vapor deposition (LPCVD). Etch windows were then transferred to this thin film via lithography and CF_4_ reactive ion etching (RIE). KOH at 89°C provided the anisotropic etching through these windows to form the cavities. One of the four layers depicted in Figure 3 housed the microlens diaphragm, whose process was described in the preceding section. The bottom most layer contained the MEMS actuator, also processed separately. The four layers were aligned and bonded in the next step.

After removing nitride diaphragms via ultrasonication, the wafers were handled within a contact aligner (MJB3, Karl-Suss) for aligned epoxy bonding. One wafer was fixed onto a glass plate by capillary force with controlled amount of water. The other wafer was then spun with premixed epoxy and quickly transferred to the aligner for aligning and contact bonding. When the wafers were aligned, they were brought to contact by physical force and securely seated. In addition to this aligned bonding, the layer containing silicon micromirror was coated with evaporated aluminum (1,000 Å) to enhance reflectivity, with oxygen plasma cleaning to remove excess epoxy.

This bonded optical subsystem was diced to individual unit dies of 5 mm by 5 mm. However, dicing was carried out to leave 80 μm of thickness to allow wafer handling prior to encapsulating the MEMS components. The MEMS components were partially diced just as in packaging, released via RIE in CF4 plasma, and align bonded to the SSC encapsulation with epoxy. The resultant wafer level package could be separated by breaking the partially diced wafers by hand (Figure 3b).

## 3. Microlens Characterization by Gaussian Beam Propagation

Microlens focusing is a Gaussian transformation. When combined with the resolving power of the microspectrometer's grating, it produces a linear dispersion that critically affects the overall system resolution. To measure the focusing performance, six microlens structures with varying focal lengths were fabricated and characterized. The Gaussian beam focusing was employed as the main method of characterization, in which the microlens focused and transformed a near-Gaussian input (633 nm HeNe) into a focal point.

**Figure 4.**
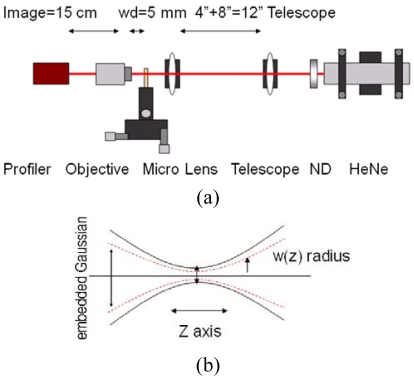
(a) Schematic of beam profiling with a CCD camera. (b) Focusing of Gaussian beam (dotted line) and non-Gaussian beam (solid line).

In the setup (Figure 4a), the laser beam was first reduced through a telescope to halve the diameter to 0.6 mm to cover the central portion of the 1.2 mm microlens diameter. As the beam was focused through the lens to micrometer diameters, a 25× microscope objective was used to image the beam waists/cross-sections onto a beam profiler charged coupled detector, CCD (Analog BeamView Analyzer, Coherent). This measured a trend of beam diameters versus focal axis position. The Gaussian propagation theory expresses the width of the focus as a function of position *z* along the focal direction as in equation (1), where *w_0_* is the minimum focal width and M^2^ is a fitting factor that translates into how well the lens acts as a Gaussian focusing element. Typical high quality lens transforms a Gaussian beam and produces focusing that closely matches the theoretical equation with a M^2^ value of 1.2 to 1.3 [15,16]. Equation (2) is the theoretical focal width of the Gaussian propagation, where *w_g_* is the diffraction limited width while *λ, f*, and *D* are the wavelength, focal length, and beam aperture, respectively.

(1) $$w^{2}(z)={w_{0}}^{2}+\frac{\lambda^{2}M^{2}}{\pi^{2}{w_{0}}^{2}}{\left(z-z_{0}\right)}^{2}$$

(2) $$2w_{g}≅2\frac{\lambda f}{D}$$

**Figure 5.**
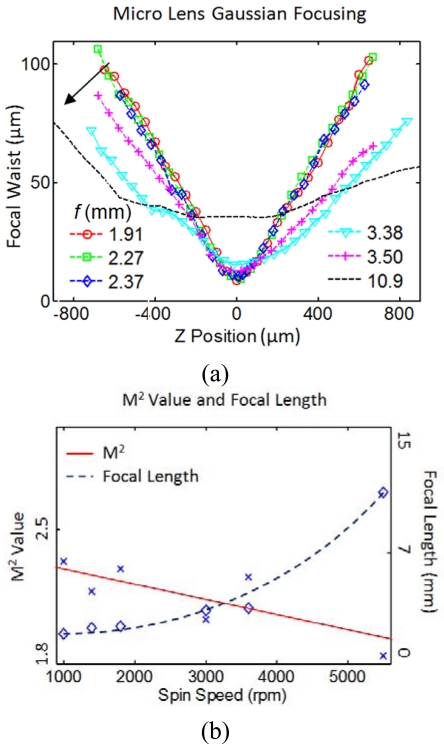

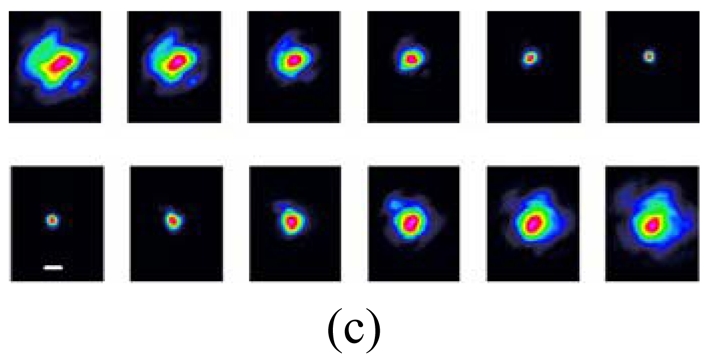
(a) Measured radii along the focal axis of six microlens diaphragms with arrow showing increasing focal length (*f* in mm). (b) M^2^ value is plotted on the left scale, while focal length is plotted on the right scale. Higher speed on the horizontal axes yields thinner initial resist, with a fixed 1.2 mm diameter, higher speed also yields lower aspect ratio. Where both curves cross indicates a balance of lens M^2^ quality and 2 mm focal length required in our spectrometer. (c) Sequence of intensity profiles as the cross sections of the focal axis are taken with 50 μm steps, scale bar 10 μm (for M^2^ 1.98, *f* 3.5 mm lens).

A set of six microlens diaphragms with 1.2 mm diameter was fabricated using double layers of 14, 15.5, 17, 20, 24, and 35 μm resist, yielding total initial thicknesses of 28, 31, 34, 40, 48, and 70 μm before reflow, respectively. The fabricated lenses were measured via Gaussian focusing and their beam profiles are presented in Figure 5a. M^2^ values are shown in Figure 5b on the left scale. In the same plot measured focal length is plotted on the right scale and matches values calculated by volume conservation during reflow. Figure 5c shows the actual images of the beam cross-sections.

The M^2^ value measured from 2.5-1.8 and decreased with increasing spin rate (thinner lens, thus lower aspect ratio), suggesting that decreasing initial thickness and aspect ratio promote accurate reflow curvature. On the other hand, though better M^2^ (1.77 at 5,500 rpm) were attained by thinner lenses, their yield during reflow was lower and produced much longer focal lengths. Other studies [17,18] also show that thinner resist creates deformation during the reflow and suggest the aspect ratio to be a critical parameter in lens formation. Balancing quality and short focus, the microlenses in our MEMS spectrometer are typically fabricated at 3000 rpm speed with initial resist thickness of 34 μm. With this process, the best fabricated microlens obtained an M^2^ of 1.98 with *f* of 3.5 mm.

## 4. System Response and Dispersion of Packaged Microlens

**Figure 6.**
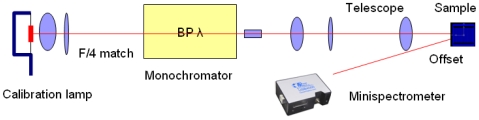
Spectroscopic measurement of efficiency with a custom tunable calibration source.

While Gaussian beam propagation analysis described the standalone microlens performance, the performance of the microlens integrated system was measured by its system response versus input wavelengths. For clarity, the MEMS grating was not actuated as the microspectrometer scanning will be reported elsewhere. To tune the input for system response, we use a customized light source: a calibration lamp (Calibration Lamp 63358, Oriel) was coupled into a monochromator (SP-2300i, Princeton Instruments) with *f*/4. The output was collimated and its diameter squeezed by a 12x telescope to a final beam of 2 mm. This tunable collimated beam was apertured to 0.5 mm for coupling into the microspectrometer, and the output was measured at the first order with a spectrometer (HR4000, Ocean Optics), Figure 6. Moreover, we characterized the separation of wavelength by coupling a dual wavelength HeNe laser, at 543 and 594 nm, directly into the system and imaging the diffraction on a CCD beam profiler used earlier in the Gaussian testing.

**Figure 7.**
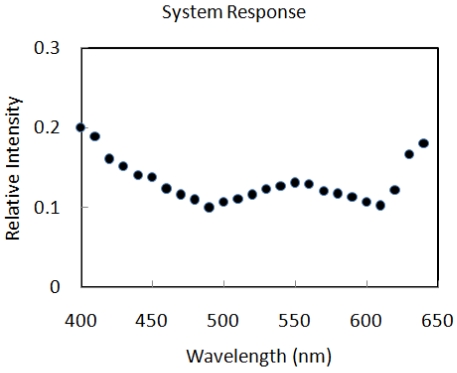
System response of the packaged microspectrometer. The 540 nm response has efficiency of 15%, for reference, cured SU8 transmission in the visible is above 95%.

The system response of the microlens integrated microspectrometer is shown in Figure 7. Overall system response contains the grating response (250 l/mm, rectangular groove depth 350 nm) as well as the dispersive performance of the microlens and the optical packaging. A 15% relative intensity (grating to blank Al) was measured for the microspectrometer at 540 nm. The prototype microspectrometer was not optimal in terms of the grating as well as the spectroscopic geometry to obtain higher system response. In addition, much noise in the system response was qualitatively observed and attributed to straylight introduced by the packaging (i.e. misalignments, unintended reflections, etc.).

**Figure 8.**
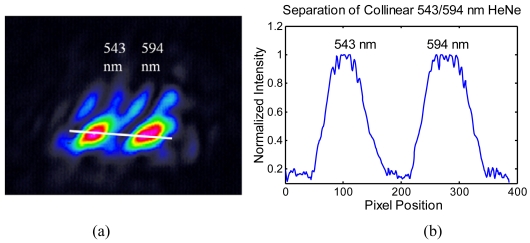
(a) Separation of collinear laser sources at 543 nm and 594 nm. (b) The intensity profile along line drawn in (a), providing resolution and straylight interpretations.

Next, two wavelengths from a dual line, collinear HeNe laser (Model LHGYR-0020, PMS Electrooptics) were separated by the microlens integrated spectrometer. The image (Figure 8) was taken 5 cm from the device with the CCD profiler used in Gaussian analysis. The 543 and 594 nm lines were clearly separated. Intensity profile given in Figure 8b shows that the two wavelengths have pixel separation (∼175 pixels) that is roughly 2-3 times their full width at half maximum values (60, 80 pixels). This suggest at least one more peak can be resolved between the two wavelengths, meaning three wavelengths ∼25 nm wavelengths apart, i.e. 543/570/594 nm, can be separated at their full width half max points. As the resolution is influenced by the grating resolving power (250 l/mm), the separation can be improved by upgrading the grating.

## 5. Conclusions

This paper described the fabrication and characterization of a diaphragmed microlens as integrated in a microoptic spectrometer built for fluorescence biosensing. This microspectrometer represents a unique application towards miniaturized time-resolved fluorescence detection. A measurement scheme was designed to measure the microlens focusing via Gaussian beam focusing analysis, with the most robust lens M^2^ of 1.98. Moreover, a custom tunable source was constructed from a calibration lamp, providing a stable source for measuring spectral efficiency of the integrated system. An efficiency of 15% was measured, hinting to un-optimized geometry. A spectral separation with 25 nm resolution was demonstrated with a collinear HeNe laser.

In the Gaussian beam propagation, it was seen experimentally that for our microlens geometry, the process has a trade-off point for focal lengths in 2-3.5 mm with M^2^ around 2. This can be explained by the film thickness as the photoresist reflows. Thinner resist (i.e. 24 μm thickness) for a given lens diameter (1.2 mm) means lower aspect ratio to reflow. Thicker resist (i.e. 70 μm thickness) on the other hand has a higher aspect ratio for the same lens diameter (1.2 mm). The photoresist has specific surface tension properties and thus a particular contact angle with the Si_x_N_y_ coated surface of the lens master wafer during reflow. Because of this, a particular range of aspect ratio should be more compatible with this contact angle and results in better spherical profile after reflow.

The system response and dual laser measurements demonstrated the efficiency and wavelength separation performance of the microlens integrated microspectrometer. As this version of the microspectrometer is limited by the geometry of fixed silicon cavity packaging, future versions will have geometry matching the MEMS gratings in order to optimize the efficiency and minimize the straylight. The measurements shown here present a testing platform for fault finding and future verification of microspectrometer performance optimizations.

Gaussian beam propagation and spectroscopic measurements used here demonstrated new schemes for characterizing microlens and microoptics systems. With a growing list of devices and applications for microoptics, an increasing list of measuring and quantifying techniques are necessary. MEMS and microoptics devices represent special tools that bridge the microscaled Biology with macroscaled measurement devices; they also require specialized optical platform that bridges its microscaled dimensions with macroscaled characterization methods. The utility of these optical optimization techniques will be important in creating future generations of microoptics devices.
